# Ginsenoside Rg3 attenuates sepsis-induced injury and mitochondrial dysfunction in liver via AMPK-mediated autophagy flux

**DOI:** 10.1042/BSR20170934

**Published:** 2017-08-31

**Authors:** Wei Xing, Lei Yang, Yue Peng, Qianlu Wang, Min Gao, Mingshi Yang, Xianzhong Xiao

**Affiliations:** 1 Department of Intensive Medicine, Third Xiangya Hospital, Central South University, Changsha 410013, P.R. China; 2 Department of Pharmacy, First Affiliated Hospital of Hunan University of Traditional Chinese Medicine, Changsha 410013, P.R. China; 3 Department of Pathophysiology, Xiangya School of Medicine, Central South University, Changsha 410078, Hunan Province, P.R. China

**Keywords:** Autophagy, Liver injury, Mitochondrial dysfunction, Rg3, Sepsis

## Abstract

Sepsis-led mitochondrial dysfunction has become a critical pathophysiological procedure in sepsis. Since ginsenosides have been applied in the treatment of mitochondrial dysfunction, ginsenoside Rg3 was employed to study its effects on the mitochondrial dysfunction induced by sepsis. The apoptosis rate, oxygen consumption rate (OCR), reactive oxygen species (ROS), antioxidant glutathione (GSH) pools, and mitochondrial transmembrane potential (MTP) were determined in LPS-induced sepsis hepatocytes treated with different concentrations of Rg3. Then, the protein expression levels of mitochondrial biogenesis related transcription factors, autophagy-related proteins, and AMP-activated protein kinase (AMPK) signal pathway related proteins were determined by Western blotting in both *in vitro* and *in vivo* sepsis models. Rg3 shows functions of promotion of OCR, attenuation of ROS, and maintenance of GSH pools, and its conjugating activity in the *in vitro* sepsis models. Rg3-treated cells were observed to have a higher MTP value compared with the LPS only induced cells. Moreover, Rg3 treatment can inhibit mitochondrial dysfunction via increasing the protein expression levels of mitochondrial biogenesis related transcription factors. Rg3 treatment has the function of inhibitor of apoptosis of human primary hepatocytes, and Rg3 can up-regulate the autophagy-related proteins and activate AMPK signal pathway in sepsis models. Meanwhile, the mitochondrial protective function exerted by Rg3 decreased after the autophagy inhibitors or AMPK inhibitor treatment in LPS-induced human primary hepatocytes. Rg3 can improve mitochondrial dysfunction by regulating autophagy in mitochondria via activating the AMPK signal pathway, thus protecting cell and organ injuries caused by sepsis.

## Introduction

Sepsis is a kind of systemic inflammatory response syndrome (SIRS) caused by microorganisms like bacteria and viruses, which in severe cases can induce septic shock and multiple organ dysfunction syndrome (MODS), and therefore it has been one of the cardinal reasons for the death of critical patients [1]. The high morbidity of sepsis keeps it a major problem to be solved currently, so more and more attention has been paid to its pathogenesis. Although much progress has been made in the early treatment of those with septic shock, people know little about the pathogenesis of organ dysfunction triggered by septicemia. Sepsis features the activation of systemic inflammatory pathway within blood vessels, by which potent inflammatory mediators are released into the blood, thus leading to septic shock, multiple organ dysfunction, adverse clinical outcomes, and high mortality [1,2]. As an important visceral organ, liver has multiple metabolic functions, such as composition, decomposition, detoxication, and immunity, and during sepsis-induced MODS, it is one of the important organs most frequently implicated by sepsis [3,4]. Under the state of sepsis, the mitochondria of important organs will see dysfunction, and the severity of mitochondrial dysfunction corresponds to the badness of prognosis which includes hemodynamic instability, tissue ATP decrease, and significant mortality increase [4]. The pathogenesis of sepsis inducing mitochondrial dysfunction are not clear yet. Therefore, a better understanding of the mechanism of mitochondrial dysfunction during severe sepsis can provide a new direction for the clinical treatment of sepsis. Studies proved that mitochondrial dysfunction is an early manifestation of liver dysfunction [4,5]. Acute liver injury induced by sepsis is largely affected by mitochondrial dysfunction and cellular energy depletion [4,5]. The mechanisms of acute liver injury induced by early septic shock include mitochondrial dysfunction and cell apoptosis, within which mitochondrial dysfunction is the key factor, involving oxidative stress injury, ATP generation reduction etc. [4,5].

Reactive oxygen species (ROS) is a by-product of biological oxygen metabolism and is a generic term of substances formed with oxygen in the organism [6]. Amongst them, the most important two ROSs are superoxide anion and hydroxyl radical. In most cells, more than 90% of the oxygen is consumed in mitochondria, 2% of which is converted into ROS in the inner membrane and matrix of mitochondria [6]. Oxidative stress refers to the generation of excessive active molecules like ROS after the organism is stimulated by various noxious stimulations, such as hunger, viruses, bacteria etc. [6]. The accumulation of ROS in mitochondria severely affects the normal function of mitochondria and oxidative stress can induce the mutation in mtDNA and increase lipid peroxidation levels [6]. In addition, ROS can change the mitochondrial membrane potential by changing the permeability of mitochondrial membrane and opening inner membrane anion channel [7]. Since mitochondria are not the only major places where ROS is generated, but also the most vulnerable area to be attacked by ROS; oxidative stress is closely related to mitochondrial dysfunction [6,8,9]. Mitophagy, i.e. selectively degrading mitochondria through autophagy, can delay the mitochondrial dysfunction induced by oxidative stress and reduce the accumulation of mtDNA mutations [10,11]. The regulation of autophagy is complicated which involves multiple signal pathways, including mechanistic target of rapamycin (mTOR), AMP-activated protein kinase (AMPK), nuclear factor (NF)-κB, and mitogen-activated protein kinase (MAPK)-mediated signaling pathway etc. [12–14]. The renewal of mitochondria depends on autophagy, yet the relationship amongst autophagy, ROS signal, and mitochondrial dysfunction is still not clear; but when autophagy is blocked, the aggregation of toxic protein and mitochondrial dysfunction will further aggravate oxidative stress [4,10,15].

As a herbaceous plant belonging to family Araliaceae, ginseng has been used as a drug in Asia and other regions for over 2000 years [16,17]. In recent years, more than 50 kinds of ginsenosides have been found, amongst which ginsenoside Rg3 (SPG-Rg3), as the active ingredient extracted from ginseng, is found to play an active role in the treatment of clinical diseases in recent studies [18,19]. It can not only affect biological functions in tumor cells, such as proliferation, apoptosis, invasion, migration, and angiogenesis, but can also reverse the phenomenon of multidrug resistance in tumor [18,19]. In recent studies, it is reported that ginsenosides are involved in the regulation of mitochondrial dynamics imbalance [20] and autophagy [21] so as to inhibit mitochondrial dysfunction. Therefore, in present study, we proposed that Rg3 can improve mitochondrial dysfunction by regulating autophagy in mitochondria, thus protecting cell injuries caused by sepsis.

## Materials and methods

### Cell culture and treatments

Human primary hepatocytes (Corning, U.S.A.) were cultured in Dulbecco’s modified Eagle’s medium (DMEM) (Invitrogen, U.S.A.) supplemented with 10% FBS, 100 units/ml penicillin, and 100 μg/ml streptomycin. Cells were cultured to 80% confluence and then treated with 100 ng/ml lipopolysaccharide (LPS) for 12 h. After the treatments, cells were harvested for further analyses. Stock solution (100 mM) of Rg3 (Meilunbio, China) was prepared with DMSO and diluted to the indicated final concentrations with culture medium before treatment. Human primary hepatocytes were divided into six groups: cells treated with vehicle (control group), cells treated with LPS (LPS group), cells treated with LPS and different concentrations of Rg3 (LPS + Rg3 6.25, 12.5, and 25 μM groups), and cells treated with Rg3 at 25 μM (Rg3 group).

### Animals and cecal ligation and puncture induced sepsis model

Male C57BL/6 mice (20–25g) were obtained from Animal Experimentation Center of Xiangya School of Medicine, Central South University. The rats were maintained in cages with an artificial 12-h light-dark cycle at 25°C, and fed with a standard diet and water. All the animal related experiments and procedures were approved by the Third Xiangya Hospital of Central South University. The sepsis rat models were established by cecal ligation and puncture (CLP) as previously reported [22,23]. Mice were intraperitoneally injected with Rg3 10 or 20  mg/kg body weight for 1  h before CLP. Mice in the control group were administrated with equivalent volume of vehicle (DMSO) for 1  h. Mice were killed and livers were collected at 6 h after CLP. The survival states of different groups were recorded at different intervals.

### Apoptosis assay

Apoptosis in LPS-induced sepsis hepatocytes treated with different concentrations of Rg3 was performed using FITC-Annexin V apoptosis detection assay (BD, U.S.A.) according to the manufacturer’s protocol. The apoptotic cells were estimated by flow cytometry.

### Measurement of oxygen consumption rate

Hepatocytes (20000/well) were seeded in HEPES-buffered, serum-free DMEM and plated on an XF24 cell culture microplate (Seahorse, U.S.A.). After different treatments in each group, cells were rinsed with XF assay medium (Seahorse, U.S.A.) and then incubated in a 37°C incubator without CO_2_ for 60 min. Then, XF24 plates were transferred to the XF24 instrument, and oxygen consumption rate (OCR) was measured for each group.

### ROS measurement

A DCFDA Cellular ROS Detection Assay Kit (Abcam, U.K.) was employed to measure the intracellular production of ROS according to the manufacturer’s protocol. The FACS analysis was applied to determine the ROS level in each group by detecting the fluorescence of DCFDA (535 nm) using an FACSCanto II cytometer (BD, U.S.A.).

### Determination of cellular GSH content and GST activity

The concentrations of cytosolic and mitochondrial GSH and GST in hepatocytes were measured by a GSH Colorimetric Detection Kit (Invitrogen, U.S.A.) and a GST colorimetric Activity Assay Kit (BioVision, U.S.A.) according to the manufacturer’s instructions. Lysed cell supernatants were assayed for GSH and GST measurement. The absorbance was determined at 405 nm (GSH) and 380 nm (GST) using a microplate reader.

### Mitochondrial transmembrane potential measurement

JC-1 was used to determine the mitochondrial transmembrane potential (MTP) of cells. First, hepatocytes were incubated within medium containing 10 μM JC-1 for 15 min. Then, cells were transferred to a clear 96-well plate to detect the JC-1 aggregate fluorescence (excitation at 488 nm and emission at 583 nm) and JC-1 monomer fluorescence (excitation at 488 nm and emission at 525 nm). Meanwhile, cells were cultured on the cover slips in a 24-well plate after 15-min incubation with 10 μM JC-1. Then, the coverslips were mounted on Dako Cytomation fluorescent mounting medium (Dako, U.S.A.) and visualized under a fluorescence microscope.

### Mitochondria extraction

Mitochondria Isolation Kit for Cultured Cells (Thermo Scientific, U.S.A.) was used in the present study. The mitochondrial and cytosolic fractions were separated by differential centrifugation. The isolated mitochondria were maintained on ice before downstream processing.

### Western blot analysis

Cells and freshly isolated mitochondria were washed with cold PBS and lysed in the RIPA buffer (Sigma–Aldrich, U.S.A.). Protein concentrations were determined using the BCA Protein Assay Kit (Thermo Fisher Scientific, U.S.A.). Proteins (30 μg) were subjected to SDS/PAGE (10%) and then electrotransferred on to a nitrocellulose membrane. Transferred proteins were then incubated with primary antibodies against PGC1-α, NRF1, Tfam, LC3B I, LC3B II, p62, beclin-1, AMPK, p-AMPK, acetyl-CoA carboxylase (ACC), p-ACC, OPA1, NADH ubiquinone oxidoreductase (complex I), and succinate dehydrogenase (complex II) (Abcam, U.S.A.). β-actin, α-tubulin, and VDAC1 were loaded as the internal references. Bands were visualized using the goat anti-rabbit IgG-HRP secondary antibody (1:2000; Abcam, U.S.A.). Bands were developed using chemiluminescent substance (Thermo Scientific, U.S.A.).

### Statistical analysis

All data were expressed as the means ± S.D. One-way ANOVA with multiple comparisons using Dunnett’s test was applied to compare the differences amongst the groups. *P*<0.05, *P*<0.01, and *P*<0.001 were considered significantly different.

## Results

### Effect of Rg3 on LPS-induced apoptosis in sepsis model

**Figure 1:**
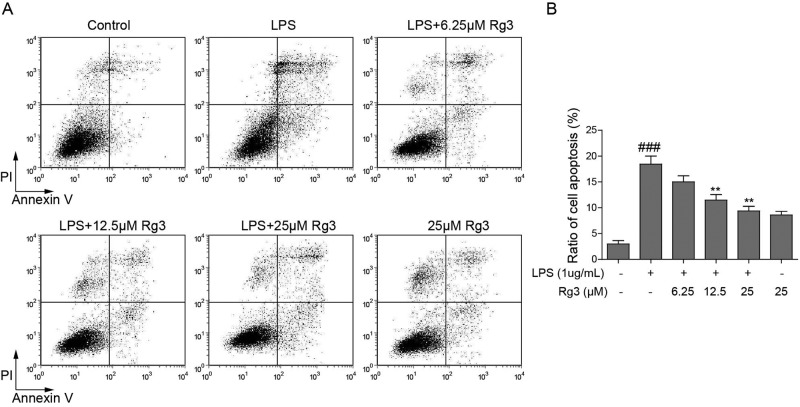
Rg3 inhibits LPS-induced apoptosis in sepsis model (**A**) Representative dot plots of apoptosis rate measured by flow cytometry. (**B**) Quantitative analysis of % apoptotic death. ^###^, *P*<0.001, compared with the control group. ###, *P* <0.001; **, *P*<0.01, compared with LPS group.

LPS-treated human primary hepatocytes served as an *in vitro* sepsis model in the present study. Human primary hepatocytes pretreated with vehicle or Rg3 (6.25, 12.5, and 25 μM) for 6 h and then underwent 24-h LPS treatment. As shown in Figure 1A,B, a significant increase in apoptotic rate of hepatocytes was observed after treatment with LPS alone (*P* <0.001 compared with the control group), while Rg3 (6.25, 12.5, and 25 μM) pretreatment reduced the apoptotic rate in a dose-dependent manner. Rg3 at 12.5 and 25 μM significantly reduced the apoptotic rate in comparison with the LPS group (*P* <0.01). These results indicate that Rg3 treatment has the function of inhibitor of apoptosis in an *in vitro* sepsis model.

### Rg3 attenuates the LPS-induced ROS production and promotes OCR in sepsis model

**Figure 2:**
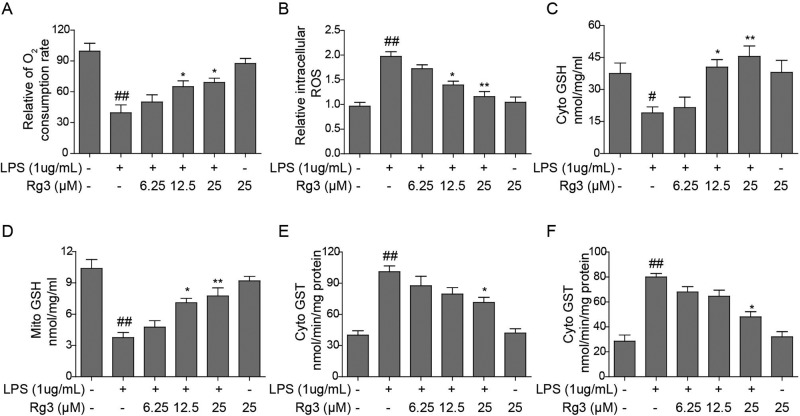
Effects of Rg3 on OCR, ROS production, and antioxidant GSH pools in sepsis model Rg3 promotes OCR (**A**), attenuates the LPS-induced ROS production (**B**), and maintain antioxidant GSH pools (**C**,**D**) and its conjugating activity (**E**,**F**) in a dose-dependent manner. ^##^, *P*<0.01, compared with the control group; *, *P*<0.05; **, *P*<0.01, compared with the LPS group.

The effects of Rg3 on the ROS production and OCR in sepsis models were studied. First, the influence of LPS *in vitro* on OCR and ROS production were determined in hepatocytes. The results showed that OCR was significantly decreased and ROS production was promoted in hepatocytes that underwent LPS treatment (*P*<0.01 compared with the control group; Figure 2A,B). Then, a promotion and an attenuation pattern of OCR and ROS production was observed in the *in vitro* sepsis models with Rg3 treatments, respectively, and Rg3 at 12.5 and 25 μM showed significant effects in comparison with the LPS group (*P*<0.05).

### GSH-dependent redox metabolism in LPS-induced sepsis model with Rg3 treatment

A significant decrease in GSH concentration was observed in both the cytosolic (*P*<0.05 compared with the control group) and mitochondrial (*P*<0.01 compared with the control group) fractions of hepatocytes treated with LPS (Figure 2C,D). Moreover, a protective effect was observed in the Rg3-treated groups in a concentration-dependent manner. Furthermore, the GSH–CDNB conjugating activity by GST enzyme was detected. LPS treatments significantly increased the GST activity in the cytosolic as well as the mitochondrial fractions (*P*<0.01 compared with the control group; Figure 2E,F). Consistently, a protective effect was observed in the Rg3-treated groups in a concentration-dependent manner (Figure 2E,F). These results indicate that the Rg3 shows a function of maintenance of antioxidant GSH pools and its conjugating activity in the *in vitro* sepsis models.

### Rg3 inhibits LPS-induced mitochondrial dysfunction in sepsis model

**Figure 3:**
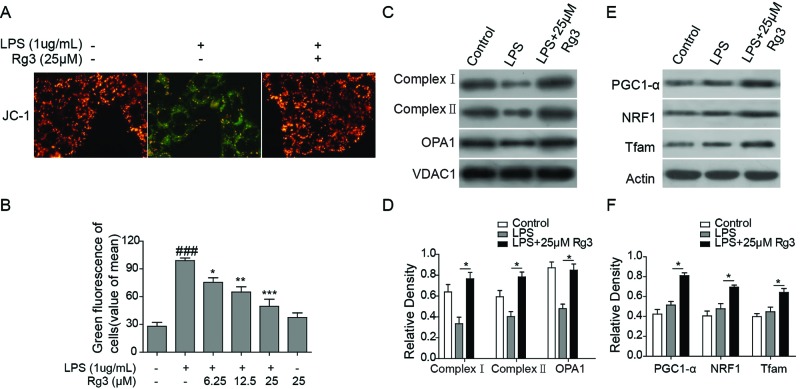
Rg3 inhibits LPS-induced mitochondrial dysfunction in sepsis model (**A**) Representative fluorescence microscopy images of JC-1. LPS-treated human primary hepatocytes were observed to have JC-1 monomer form (cells with green fluorescence), indicating lower MTP. However, both the negative control cells and Rg3-treated cells were observed to have JC-1 aggregate form (cells with red fluorescence), indicating high MTP values. (**B**) Quantitative analysis of JC-1 fluorescence. (**C**,**D**) Expression levels of respiratory chain complexes assembly proteins in mitochondria determined by Western blotting. the expression levels of OPA1, complex I, and complex II were down-regulated with LPS treatment, which were reversed by the Rg3 treatment. (**E**,**F**) Expression levels of mitochondrial biogenesis related transcription factors PGC1-α, NRF-1, and Tfam-1 as determined by Western blotting. *, *P*<0.05; **, *P*<0.01, ***, *P*<0.001, compared with the LPS group.

In order to investigate whether Rg3 conferred a protective effect on mitochondrial damage caused by sepsis, MTP was measured in an LPS-induced *in vitro* sepsis model. As reported, an increased level of JC-1 monomers indicates a low MTP, and an increased level of J-aggregate form indicates a normal MTP [21]. Our fluorescence microscopy images showed that LPS-treated human primary hepatocytes were observed to have JC-1 monomer form (cells with green fluorescence), indicating lower MTP. However, both the negative control cells and Rg3-treated cells were observed to have JC-1 aggregate form (cells with red fluorescence), indicating high MTP values (Figure 3A,B). Further, the expression levels of respiratory chain complexes assembly proteins in mitochondria were determined. As shown in Figure 3C,D, the expression levels of OPA1, complex I, and complex II were down-regulated with LPS treatment, which were reversed by the Rg3 treatment (*P*<0.05 for complex I and complex II compared with the LPS only treated group). These results indicate that Rg3 alleviated the mitochondrial dysfunction induced by LPS *in vitro*.

### Rg3 inhibits mitochondrial dysfunction via restoration of mitochondrial biogenesis

It has been reported that sepsis can induce the increase in mitochondrial biogenesis [24,25]. Therefore, CLP-induced sepsis rat model was employed to further study the effect of Rg3, and the influence of LPS on mitochondrial biogenesis was determined. Treatment of hepatocytes with LPS resulted in increased expression of the mitochondrial biogenesis related transcription factors PGC1-α, NRF-1, and Tfam-1, as determined by Western blotting (Figure 3E,F). LPS induction of these transcription factors was also confirmed in whole-liver homogenates following CLP (Figure 6B,C). Moreover, Rg3 (25 μM or 20 mg/kg) treatment on hepatocytes (Figure 3E,F) or intraperitoneal administration on rats (Figure 6B,C) further increased LPS or CLP-induced increase in mitochondrial biogenesis associated transcription factors, as demonstrated by Western blotting in both *in vitro* and *in vivo* sepsis models (*P*<0.05 compared with the LPS only treated group).

### Rg3 increases the CLP-induced sepsis rat survival rate

In order to further confirm the protective effect of Rg3 on sepsis model, the survival rate in CLP-induced sepsis rat was studied with or without Rg3 treatment. As shown in Figure 6A, Kaplan–Meier curves were applied to evaluate the rat survival rate. As expected, rats treated with 10 and 20 mg/kg Rg3 exhibited a significantly higher survival rate 72 h post-CLP induction compared with the CLP only group.

### Rg3 recovers LPS- and CLP-induced mitochondrial dysfunction in sepsis models via autophagy flux

**Figure 4:**
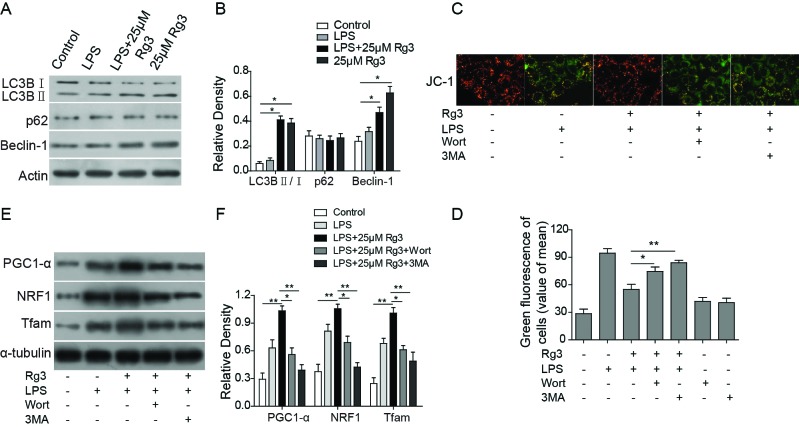
Rg3 recovers LPS- and CLP-induced mitochondrial dysfunction via autophagy *in vitro* (**A**,**B**) Expression levels of autophagy-related proteins determined by Western blotting. LC3B II/LC3B I and Beclin-1 levels were higher in Rg3-treated group than in the control group and the LPS-treated group, while Rg3 treatment showed no effect on the p62 levels. (**C**) Representative fluorescence microscopy images of JC-1. The green fluorescence in LPS-treated human primary hepatocytes was increased following exposure to autophagy inhibitors. When combined with the autophagy inhibitors, Rg3 treatment cannot inhibit the LPS-induced JC-1 monomer production. (**D**) Quantitative analysis of JC-1 fluorescence. (**E**,**F**) Expression levels of mitochondrial biogenesis related transcription factors PGC1-α, NRF-1, and Tfam-1, as determined by Western blotting. When combined with the autophagy inhibitors, expression levels of mitochondrial biogenesis related transcription factors PGC1-α, NRF-1, and Tfam-1 cannot be up-regulated even when treated with 25 μM Rg3. *, *P*<0.05; **, *P*<0.01, the groups of comparison were indicated in data.

The up-regulation in mitophagy in sepsis may play a critical role in removing dysfunctional mitochondria, and mitochondrial biogenesis may account for this restoration of mitochondrial density [24,25]. LC3B I, LC3B II, p62, and Beclin-1 are central autophagy related proteins involved in the autophagy flux [26]. Therefore, whether Rg3 unregulated these autophagy-related proteins was studied both *in vitro* (Figure 4A,B) and *in vivo* (Figure 6D,E) sepsis models. We identified that the LC3B II/LC3B I and Beclin-1 levels were higher in Rg3-treated group than in the control group or sham group and the LPS- or CLP-treated group, while Rg3 treatment showed no effect on the p62 levels. These findings suggest that Rg3 could activate autophagy in both *in vitro* and *in vivo* sepsis models.

Furthermore, in order to confirm whether Rg3 has a mitochondrial protective function via activating autophagy, the autophagy inhibitors, 3-methyladenine (3MA) and wortmannin (wort) were applied, and the MTP examined. The results showed that the green fluorescence in LPS-treated human primary hepatocytes was increased following exposure to autophagy inhibitors. When combined with the autophagy inhibitors, Rg3 treatment cannot inhibit the LPS-induced JC-1 monomer production (*P*<0.05 compared with the LPS + Rg3 group; Figure 4C,D). Meanwhile, when combined with the autophagy inhibitors, expression levels of mitochondrial biogenesis related transcription factors PGC1-α, NRF-1, and Tfam-1 cannot be up-regulated even when treated with 25 μM Rg3 (Figure 4E,F). These indicate that the mitochondrial protective function exerted by Rg3 decreased after the autophagy inhibitor treatment in LPS-induced hepatocytes.

### Rg3 promotes mitochondrial autophagy in sepsis models via AMPK signal pathway activation

**Figure 5:**
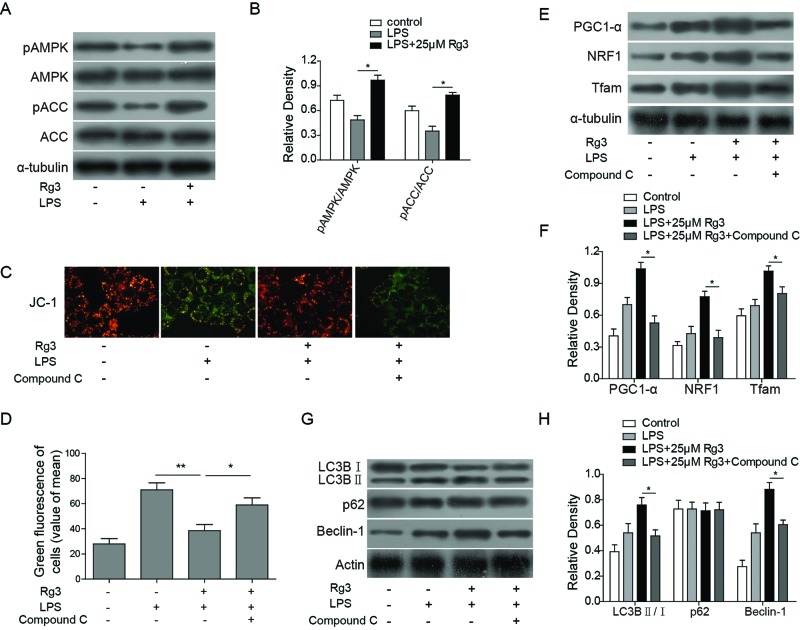
Rg3 promotes mitochondrial autophagy via AMPK signal pathway activation *in vitro* (**A**,**B**) Expression levels of AMPK signal pathway related proteins determined by Western blotting. LPS treatment decreased the levels of p-AMPK and ACC (p-ACC) in hepatocytes, while the Rg3 treatment reversed the inhibition effect brought by LPS, further promoting the phosphorylation of the AMPK signal pathway related proteins. (**C**) Representative fluorescence microscopy images of JC-1, the green fluorescence in Rg3-treated, LPS-induced hepatocytes was increased, while it decreased when exposed to Compound C. (**D**) Quantitative analysis of JC-1 fluorescence. (**E**,**F**) Expression levels of mitochondrial biogenesis related transcription factors determined by Western blotting. The expression levels of PGC1-α, NRF-1, and TFAM-1 were down-regulated when treated with Compound C compared with the LPS + Rg3 group. (**G**,**H**) Expression levels of autophagy-related proteins determined by Western blotting. Compound C treatment reversed the up-regulation effects of autophagy-related proteins brought by Rg3 treatment. *, *P*<0.05; **, *P*<0.01, the groups of comparison were indicated in data.

**Figure 6:**
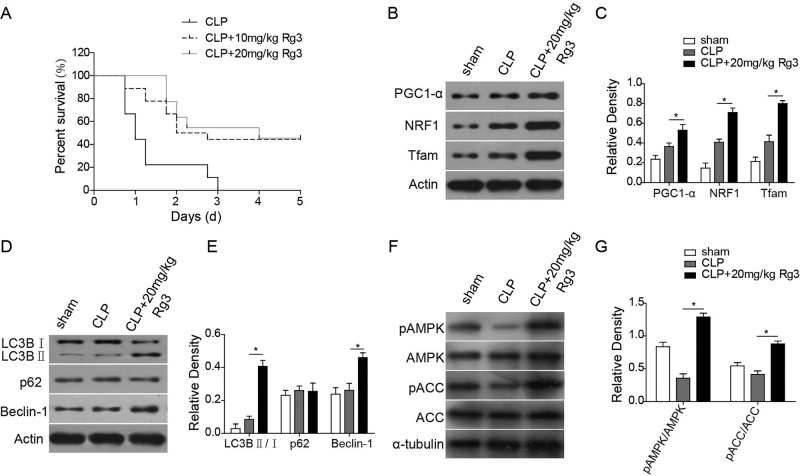
Rg3 promotes mitochondrial autophagy via AMPK signal pathway activation *in vivo* (**A**) Kaplan–Meier curves of rat survival rate. Rats treated with 10 and 20 mg/kg Rg3 exhibited a significantly higher survival rate 72 h post-CLP induction compared with the CLP only group. (**B**,**C**) Expression levels of mitochondrial biogenesis related transcription factors PGC1-α, NRF-1, and Tfam-1, as determined by Western blotting. (**D**,**E**) Expression levels of autophagy-related proteins determined by Western blotting. LC3B II/LC3B I and Beclin-1 levels were significantly up-regulated in Rg3-treated group than in the sham group and the CLP-treated group, while Rg3 treatment showed no effect on the p62 levels. (**F**,**G**) Expression levels of AMPK signal pathway related proteins determined by Western blotting. CLP treatment decreased the levels of p-AMPK and ACC (p-ACC) in rat models, while the Rg3 treatment reversed the inhibition effect brought by CLP treatment, further promoting the phosphorylation of the AMPK signal pathway related proteins. *, *P* <0.05, compared with the CLP only group.

AMPK signal pathway plays a critical role in cellular processes such as autophagy [27]. Therefore, the AMPK signal pathway related proteins were studied and an AMPK inhibitor, Compound C, was recruited in the present study. Our results (Figure 5A,B) showed that LPS treatment decreased the levels of p-AMPK and ACC (p-ACC) in hepatocytes, which indicates that LPS treatment reduces the activation of AMPK signal pathway. However, the Rg3 treatment reversed the inhibition effect brought by LPS, further promoting the phosphorylation of the AMPK signal pathway related proteins. Furthermore, Rg3 activation of AMPK signal pathway was also confirmed in whole-liver homogenates following CLP *in vivo* (Figure 6F,G). In order to further confirm whether Rg3 can promote mitochondrial autophagy in sepsis models via AMPK signal pathway, the MTP, mitochondrial biogenesis related transcription factors, and central autophagy related proteins were examined after the treatment of AMPK inhibitor Compound C in LPS-induced hepatocytes. The results showed that the green fluorescence in Rg3-treated, LPS-induced hepatocytes was increased (*P*<0.01 compared with the LPS only group; Figure 5C,D), while it decreased when exposed to Compound C (*P*<0.05 compared with the LPS + Rg3 group; Figure 5C,D). Also, for the mitochondrial biogenesis related transcription factors, the expression levels of PGC1-α, NRF-1, and TFAM-1 were down-regulated when treated with Compound C compared with the LPS + Rg3 group, which indicates that AMPK inhibitor can reverse the protective effect of Rg3 on mitochondrial biogenesis (Figure 5E,F). Furthermore, Compound C treatment also reversed the up-regulation effects of autophagy-related proteins brought by Rg3 treatment (Figure 5G,H). All these results indicate that the Rg3 exerts mitochondrial protective function via activation of AMPK signal pathway, then further promotes mitochondrial autophagy in sepsis models.

## Discussion

Sepsis is a kind of life-threatening organ dysfunction caused by infection-induced body imbalance [4]. Many studies have shown that sepsis can further develop into severe sepsis, septic shock and MODS, and even cause death [4,8]. Statistics reveal that there are ~750000 new cases of patients with severe sepsis per year in the United States with a yearly increase rate of 1.5% and there will be millions of new severe sepsis patients per year up to the year 2020 [28]. Unfortunately, until now there are still no acknowledged effective specific medicines widely used in clinics to treat sepsis. The pathophysiological procedures of sepsis mainly include cytokine storm, inflammatory mediator waterfall, bacterial translocation and intestinal endotoxemia, interaction between coagulation system and inflammatory system, as well as microcirculation and mitochondrial dysfunction etc. [4,29]. In recent years, sepsis-led mitochondrial dysfunction has become a research hotspot [4].

Sepsis leads to excessive oxidative stress during which excessive ROS – the key factor causing the structural damage and dysfunction of mitochondria – are generated. The change in mitochondrial membrane potential is an important indicator reflecting the structural damage and dysfunction in mitochondria [6]. The decrease or disappearance in mitochondrial membrane potential prompts the structural damage and dysfunction of mitochondria. In the present study, we found that ROS production was significantly increased in hepatocytes that underwent LPS treatment (*P*<0.01 compared with the control group). Then, an attenuation pattern of ROS production was observed in this *in vitro* sepsis model with Rg3 treatment. Also, Rg3-treated cells were observed to have a higher MTP value compared with the LPS only induced cells. It has been reported that the change in membrane potential may also lead to ATP production capacity to decrease or disorder, which further results in excessive production of ROS, finally causing structural damage to mitochondria and even the damage to the entire cell [4,8]. Therefore, we proposed that Rg3 may increase the ATP production capacity to alleviate the mitochondrial dysfunction induced by LPS.

As a vital organ of the body for substance and energy metabolism, liver is not only an important place where cytokines are generated, but also one of the most vulnerable organs with the most violent inflammatory reactions during the occurrence and development of sepsis [5,30]. The incidence of liver injury induced by sepsis is as high as 34.7%, and meanwhile liver dysfunction is one of the signs of sepsis developing into multiple organ dysfunctions and is the earliest syndrome of MODS occurrence with the most remarkable damage [30]. In the present study, whole-liver homogenates from rats that underwent CLP and human primary hepatocytes that underwent LPS treatment were employed to serve as *in vivo* and *in vitro* sepsis models, respectively. When sepsis occurs, bacteria and endotoxins can activate liver cells and induce the excessive release of ROS and massive production of ROS, leading to mitochondrial damage in the liver, liver cells’ energy supply decrease and metabolism failure, and thus failing to maintain normal functions [5,30]. Also, it has been previously demonstrated that biogenesis is increased in the setting of sepsis [24,25]. Therefore, the effect of Rg3 on the mitochondrial biogenesis associated transcription factors was studied. Our finding indicates that Rg3 treatment can inhibit mitochondrial dysfunction via restoration of mitochondrial density through increasing mitochondrial biogenesis associated transcription factors in both *in vitro* and *in vivo* sepsis models.

On the other hand, in the present study, Rg3 was also found to have the the function to remove the dysfunctional mitochondria through increasing the mitophagy in sepsis. Mitophagy refers to selectively degrading mitochondria by autophagy, which can retard and reduce the accumulation of mtDNA mutations [15,31]. When the body undergoes negative stimulations from outside world, the selective autophagy in mitochondria may occur [15,31]. Our study found that Rg3 treatment has the function of inhibitor of apoptosis of human primary hepatocytes. Also, Rg3 can unregulate the autophagy-related proteins and activate autophagy in both *in vitro* and *in vivo* sepsis models. Meanwhile, the mitochondrial protective function exerted by Rg3 decreased after the autophagy inhibitors treatment in LPS-induced human primary hepatocytes. Under oxidative stress, mitochondrial membrane proteins are subjected to oxidative damage or mtDNA mutations, causing excess protein synthesis and thus resulting in their misfolding and aggregation as well as MPT. In addition, MPT causes the depletion of ATP after the decoupling of oxidative phosphorylation, and ultimately leads to cell necrosis. Furthermore, MPT-induced mitochondrial swelling leads to the release of cytochrome *c* from mitochondria and activates a series of caspases, thus resulting in apoptosis [15,31]. However, under mild stimulations, the formation of MPT may promote the occurrence of autophagy, which is beneficial for the removal of damaged mitochondria [15,31]. Mitochondrial damage leads to the loss of its function, thus triggering mitophagy. Our findings indicate that Rg3 plays an important role in this way to remove damaged mitochondria and prevent ROS accumulation.

More interestingly, in the present study, Rg3 was found to promote the phosphorylation of AMPK. AMPK is an energy sensor of cells, regulating cellular energy metabolism, and its activation is conducive to rectifying metabolic disorders, making cell metabolism to be physically balanced [27]. Escobar et al. [32] reported that activation of AMPK can minimize the organ injury induced by sepsis by decreasing inflammatory cytokines and endothelial activation. Also, AMPK is an important positive regulator of autophagy [13,27]. Activated AMPK can promote autophagy by inhibiting TORC1 complex [27]. Therefore, we propose that Rg3 exerts mitochondrial protective function via activation of AMPK signal pathway, then further promotes mitochondrial autophagy in sepsis models. Furthermore, mitochondria are the key sites for fatty acid metabolism, while AMPK can affect the energy metabolism of mitochondria by influencing the activity of ACC [27,33]. In the present study, we also found that Rg3 treatment promoted the phosphorylation of ACC. ACC can catalyze the carboxylation of acetyl CoA and thus convert it into malonyl CoA, which both is the fatty acid synthesis precursor and meanwhile inhibits the oxidation of fatty acids in mitochondria [34]. AMPK can phosphorylate ACC, resulting in its decrease in activity and thus reducing the level of malonyl CoA [27,33,34]. When long chain fatty acids are transported to mitochondria for β-oxidation, mitochondrial translocators are necessarily involved, in which the activity of the key protein CPT-I is inhibited by malonyl CoA [27,33,34]. Thus, when AMPK is activated, it will cause the ACC phosphorylation on mitochondria, so as to result in the decrease in malonyl CoA level and thus release the inhibition against CPT-I, which in turn increases the amount of fatty acids entering mitochondria and stimulates β-oxidation, hence improving ATP synthesis [27,33,34]. Therefore, via activating the AMPK signal pathway, Rg3 may also increase the ATP production capacity to alleviate the mitochondrial dysfunction induced by LPS.

Mitophagy plays a significant role in maintaining cell homeostasis, but currently there are no clear elaborations on the mechanism of mammals’ mitophagy. Besides, the relationship between mitophagy and related diseases remains quite vague. For some natural antioxidants, the previous antioxidant mechanism mainly remains in eliminating or inhibiting ROS production, which fails to deeply and durably alleviate oxidative stress and is far from the effect of repairing the overall oxidative damage. In the present study, our results indicate that Rg3 exerts mitochondrial protective function via activation of AMPK signal pathway, then further promotes mitochondrial autophagy in sepsis models. AMPK can serve as a target point in Rg3 inducing mitophagy and the alleviation of oxidative stress. The specific binding of Rg3 with a certain receptor of mitophagy, if applicable, is beneficial to clarify the regulation mechanism of mitophagy and promote the research on the roles of mitochondrial quality control in the occurrence of diseases.

## Abbreviations

ACC: acetyl-CoA carboxylase
AMPK: AMP-activated protein kinase
CLP: cecal ligation and puncture
DCFDA: dichlorofluorescin diacetate
DMEM: Dulbecco’s modified Eagle’s medium
LC3B I/II: light chain 3B I/II
MODS: multiple organ dysfunction syndrome
MTP: mitochondrial transmembrane potential
NRF-1: nuclear repiratory factor 1
OCR: oxygen consumption rate
OPA1: optic atrophy 1
PGC-1α: PPAR gamma coactivator 1 alpha
Rg3: Ginsenoside Rg3
ROS: reactive oxygen species
Tfam: mitochondrial transcrption factor A
VDAC1: voltage-dependent anion-selective channel 1
